# *Bacillus subtilis* natto Derivatives Inhibit Enterococcal Biofilm Formation *via* Restructuring of the Cell Envelope

**DOI:** 10.3389/fmicb.2021.785351

**Published:** 2021-12-09

**Authors:** Yu-Chieh Lin, Chun-Yi Wu, Hung-Tse Huang, Mei-Kuang Lu, Wei-Shou Hu, Kung-Ta Lee

**Affiliations:** ^1^Department of Biochemical Science and Technology, National Taiwan University, Taipei, Taiwan; ^2^Ministry of Health and Welfare, National Research Institute of Chinese Medicine, Taipei, Taiwan; ^3^Graduate Institute of Pharmacognosy, Taipei Medical University, Taipei, Taiwan; ^4^Department of Chemical Engineering and Materials Science, University of Minnesota, Minneapolis, MN, United States

**Keywords:** probiotics, *Bacillus subtilis* natto, *Enterococcus faecalis*, biofilm, cell envelope synthesis

## Abstract

*Enterococcus faecalis* is considered a leading cause of hospital-acquired infections. Treatment of these infections has become a major challenge for clinicians because some *E. faecalis* strains are resistant to multiple clinically used antibiotics. Moreover, the presence of *E. faecalis* biofilms can make infections with *E. faecalis* more difficult to eradicate with current antibiotic therapies. Thus, our aim in this study was to investigate the effects of probiotic derivatives against *E. faecalis* biofilm formation. *Bacillus subtilis* natto is a probiotic strain isolated from Japanese fermented soybean foods, and its culture fluid potently inhibited adherence to Caco-2 cell monolayers, aggregation, and biofilm production without inhibiting the growth of *E. faecalis*. An apparent decrease in the thickness of *E. faecalis* biofilms was observed through confocal laser scanning microscopy. In addition, exopolysaccharide synthesis in *E. faecalis* biofilms was reduced by *B. subtilis* natto culture fluid treatment. Carbohydrate composition analysis also showed that carbohydrates in the *E. faecalis* cell envelope were restructured. Furthermore, transcriptome sequencing revealed that the culture fluid of *B. subtilis* natto downregulated the transcription of genes involved in the WalK/WalR two-component system, peptidoglycan biosynthesis and membrane glycolipid biosynthesis, which are all crucial for *E. faecalis* cell envelope synthesis and biofilm formation. Collectively, our work shows that some derivatives present in the culture fluid of *B. subtilis* natto may be useful for controlling *E. faecalis* biofilms.

## Introduction

Enterococci, which are Gram-positive bacteria normally present in human gastrointestinal tracts, are the second most common pathogens recovered from catheter-associated infections of the bloodstream and urinary tract and from skin and soft-tissue infections in hospitals in the United States (Paulsen et al., 2003; Arias and Murray, 2012). Among *Enterococcus* species, *Enterococcus faecalis* is the primary species responsible for human enterococcal infections (Sievert et al., 2013). Treatment of *E. faecalis* infections has become increasingly difficult because of the emergence of *E. faecalis* strains that are resistant to numerous clinically used antibiotics, such as macrolides; tetracyclines; aminoglycosides; and glycopeptides, including vancomycin, which was previously used as the antibiotic of last resort for enterococcal infections (Murray, 1997; Klevens et al., 2007; Arias et al., 2010; Frieden, 2013). Moreover, *E. faecalis* has a propensity to transfer antibiotic resistance genes to other bacteria within and across species *via* pheromone-inducible conjugative plasmid transfer, which facilitates the dissemination of antibiotic resistance (Clewell, 1990; Clewell et al., 2002).

In addition to having intrinsic resistance to multiple antibiotics and the ability to transfer antibiotic resistance *via* plasmid transfer, *E. faecalis* can readily form biofilms on a wide range of natural and artificial substrates, such as damaged heart valves, venous catheters, urinary catheters, and indwelling medical devices (Donlan and Costerton, 2002; Fernandez Guerrero et al., 2007). Biofilms are aggregates of microbes that accumulate at a solid-liquid interface and are encased in a self-produced matrix of extracellular polymeric substances (Flemming et al., 2007; Flemming and Wingender, 2010). Since the protective extracellular matrix can decrease the penetration of antibiotics, *E. faecalis* cells in biofilms can be 10 to 1,000 times more resistant to antibiotics than their planktonic counterparts (Hoyle and Costerton, 1991; Mah and O’Toole, 2001). This trait of enterococcal biofilms markedly reduces the effectiveness of current antibiotic treatments. In addition, enterococcal biofilms have been shown to serve as nidi for bacterial dissemination and as reservoirs for antibiotic resistance genes (Ch’ng et al., 2019). Taken together, the evidence indicates that the presence of *E. faecalis* biofilms can make infections with *E. faecalis* more difficult to eradicate. Therefore, there is a demand for novel, safe, and effective methods to inhibit the formation of *E. faecalis* biofilms.

In addition to discovering and developing new antibiotics, scientists have explored the possibility of preventing and treating gastrointestinal tract infections with probiotics, which are live microorganisms, such as bacteria and yeast, that can provide benefits to the host when administered in adequate amounts (Saarela et al., 2002; Kamada et al., 2013). The use of spore-forming bacteria, mostly of the genus *Bacillus*, as probiotics has attracted considerable attention from researchers in recent years (Elshaghabee et al., 2017). In comparison to commonly used non-spore-forming probiotic lactic acid bacteria, *Bacillus* species can form spores under harsh environments. This trait enables them to have higher acid tolerance and better stability during heat processing and low-temperature storage than other bacteria (Elshaghabee et al., 2017). In addition, previous studies (Piewngam et al., 2018; Tazehabadi et al., 2021) have shown that some *Bacillus* species possess the ability to inhibit the colonization and biofilm formation of pathogens *via* actions, such as interference with quorum-sensing signals, or production of antimicrobial agents, such as bacteriocin proteins. Piewngam et al. (2018) found that probiotic *Bacillus subtilis* can produce the lipopeptide fengycin for decolonization of methicillin-resistant *Staphylococcus aureus* (MRSA) in mouse feces and intestines *via* interference with *S. aureus* agr quorum-sensing signaling. Tazehabadi et al. (2021) found that two bacteriocin-producing *Bacillus* probiotic strains, *B. subtilis* KATMIRA1933 and *Bacillus amyloliquefaciens* B-1895, can inhibit the biofilm formation of several strains of the food-borne pathogen *Salmonella enterica* without killing planktonic *S. enterica* cells. Collectively, these studies support the idea that probiotic *Bacillus* species and their derivatives may have the potential to inhibit the biofilm formation ability of other pathogenic bacteria, such as *E. faecalis*.

*B. subtilis* natto is a probiotic strain isolated from natto, which is a traditional fermented soybean food in Japan with a long history of consumption (Nishito et al., 2010). In the process of steamed soybean fermentation, *B. subtilis* natto produces various derivatives, such as extracellular proteases, viscous substance γ-poly-DL-glutamic acid (γ-PGA), and antibiotics (Nishito et al., 2010; Katayama et al., 2021). Some *B. subtilis* natto derivatives may be useful to fight against *E. faecalis*. For example, we have shown in our previous studies that *B. subtilis* natto can secrete nattokinase mainly to degrade the peptide pheromone cCF10, thereby interfering with the transfer frequency of the antibiotic resistance plasmid pCF10 between *E. faecalis* bacteria (Lin et al., 2021). In this study, we further demonstrated the effects of *B. subtilis* natto supernatant on the biofilm formation ability of *E. faecalis*. In addition, we attempted to clarify the mechanisms by which *B. subtilis* natto supernatant affects the biofilm formation of *E. faecalis* using transcriptome sequencing (RNA-seq).

## Materials and Methods

### Bacterial Strains, Medium, and Growth Conditions

All bacterial strains used in this study are listed in Table 1. Wild-type *E. faecalis* OG1RF and constructed *E. faecalis* OG1RF::p23cfp that expresses a constitutive CFP were obtained from the laboratory of Professor Gary M. Dunny (University of Minnesota, United States). All *E. faecalis* strains were statically cultured at 37°C in M9 medium [3g/L yeast extract, 10g/L casamino acids, 36g/L glucose, 0.12g/L MgSO_4_, and 0.011g/L CaCl_2_ (Bandyopadhyay et al., 2016)] or in brain heart infusion broth (BD Co., United States). If needed, the antibiotic rifampicin was added at a concentration of 200μg/ml (Bandyopadhyay et al., 2016).

**Table 1:** Bacterial strains used in this study.

Strain or plasmid	Description	Source	Reference(s)
*E. faecalis* strains			
OG1RF	Rif^r^ Fa^r^	Dr. Gary M. Dunny	Dunny et al., 1978
OG1RF::p23cfp	OG1RF derivative with a constitutive CFP reporter fused to its genomic DNA	Dr. Gary M. Dunny	Barnes et al., 2017
Probiotic strains			
*B. subtilis* natto NTU-18 (BCRC 80390)		Our laboratory	Kuo et al., 2006; Kuo and Lee, 2008

*Bacillus subtilis* natto NTU-18 (BCRC 80390) isolated from a commercial product was maintained in our laboratory (Kuo et al., 2006; Kuo and Lee, 2008). *B. subtilis* natto was cultured in LB broth [10g/L tryptone, 5g/L yeast extract, and 10g/L sodium chloride (Atlas, 2010)] in orbital shakers at 37°C with shaking at 125rpm.

### Preparation of *B. subtilis* natto Cell-Free Supernatant

*Bacillus subtilis* natto supernatant was prepared using methods described in our previous study (Lin et al., 2021). In brief, overnight cultures of *B. subtilis* natto were diluted 1:100 in M9B medium (8.5g/L Na_2_HPO_4_·2H_2_O, 3g/L KH_2_PO_4_, 11.5g/L sodium acetate, and 1ml/L acetic acid were added as buffering agents to the original M9 medium) and cultured aerobically in orbital shakers at 37°C with shaking at 125rpm. After 24h of cultivation, the cultures were centrifuged (4,000×*g*, 10min) to remove all cells. Then, the supernatant was filter-sterilized through 0.22-μm filters (Pall Co., United States) and stored at 4°C.

### Cell Culture

Caco-2, a colon adenocarcinoma cell line, was purchased from the Bioresource Collection and Research Center (BCRC, Taiwan). The cells were routinely maintained in Dulbecco’s modified Eagle medium (DMEM containing 4.5g/L glucose; GeneDireX Inc., Taiwan) supplemented with 10% fetal bovine serum (FBS; Gibco, United States) and 1x penicillin-streptomycin-glutamine (GeneDireX Inc., Taiwan) and incubated at 37°C in a 95% humidity and 5% CO_2_ air atmosphere. The cells were passaged every 5–6days in 10cm^2^ cell culture dishes.

### *In vitro* Assay of Bacterial Adhesion Onto the Human Intestinal Caco-2 Cell Line

An *in vitro* bacterial adhesion assay was performed as described by Letourneau et al. with some modifications (Letourneau et al., 2011). To prepare monolayers of Caco-2 cells for the *in vitro* bacterial adhesion assay, one milliliter of cell suspension (2×10^5^ cells/ml) was seeded in three sets of duplicate wells (one for each treatment) of a 24-well plate, and the plate was incubated in a cell culture incubator until the cells were fully confluent. The cells were then washed with phosphate-buffered saline (PBS), and the culture medium was replaced with 900μl of antibiotic-free DMEM supplemented with 10% FBS; then, 100μl of *B. subtilis* natto supernatant or M9B medium was added.

Overnight cultures of *E. faecalis* cells were centrifuged, washed twice with PBS containing 2mm EDTA, and resuspended in DMEM with 10% FBS. A volume of bacterial culture corresponding to 10^6^ *E. faecalis* cells was used to inoculate Caco-2 cells. The same volume of *E. faecalis* culture was also added to a medium mixture (90% DMEM containing 10% FBS; 10% M9B medium) without Caco-2 cells to determine the total number of bacterial cells in the inoculum. The *E. faecalis* and Caco-2 cells were then cocultured at 37°C with 5% CO_2_ for 3h. After 3h of incubation, the culture medium was removed, and the infected Caco-2 cells were washed 3 times with PBS. All cells were then detached with 0.05% trypsin-EDTA for 20min at 37°C. Then, serial dilutions of these samples were plated on selective Todd Hewitt broth agar medium containing 30g/L Bacto Todd Hewitt Broth (Neogen Cor., United States), 15g/L agar and 50μg/ml rifampicin, and the adherent *E. faecalis* cells were counted.

### Autoaggregation Assay

An autoaggregation assay was performed as described by Baccouri et al. and Kaur et al. with some modifications (Kaur et al., 2018; Baccouri et al., 2019). Overnight cultures of *E. faecalis* OG1RF were diluted 1:100 in M9B medium with or without *B. subtilis* natto supernatant treatment (0, 10, and 50% v/v) and grown in culture tubes with screw caps and rubber liners (Kimble Inc., United States). All culture tubes were incubated anaerobically at 37°C for 24h. After 24h of cultivation, treated and untreated *E. faecalis* cultures were centrifuged, washed twice with PBS, and resuspended in PBS to give final OD_600_ of 1. The *E. faecalis* suspensions were vortexed for 15s and then incubated at 37°C for 4h. After 0 and 4h of incubation without mixing, one milliliter of the suspension from the top of the tube was taken to measure the absorbance (A) at 600nm. Autoaggregation was then calculated as follows: autoaggregation (%)=[1−(A_4h_/A_0_)×100].

### Experimental Setup for SEM

Overnight cultures of *E. faecalis* OG1RF were diluted 1:100 in a 24-well plate containing 1ml of medium and sterile cover glasses and anaerobically cultured at 37°C for 24 or 48h. The biomass that adhered to the cover glass was prefixed with 2.5% glutaraldehyde and 2.5% paraformaldehyde in 0.05M cacodylate at 25°C for 60min.

After prefixation, the samples were washed three times with 0.05M cacodylate and then postfixed with 1% osmium tetroxide in 0.05M cacodylate at 25°C for 60min. The fixed samples were chemically dehydrated using a graded ethanol series [30, 50, 70, 85, 90, 95, and 100% (two times)] and processed in a CO_2_-based critical point dryer and ion coater. The dried samples were observed using an FEI Inspect S scanning electron microscope at a high voltage of 15kV and a magnification of 3,000×.

### Biofilm Growth

A ninety-six-well polystyrene plate-based bacterial biofilm formation assay was performed as described by Dale et al. with some modifications (Dale et al., 2015). In brief, overnight cultures of *E. faecalis* were diluted 1:100 in M9B medium containing 10% *B. subtilis* natto supernatant or not, and 100μl was dispensed into 8 wells per treatment. In addition, Oxyrase^®^ for Broth (Oxyrase Inc., United States) was added to the cultures to generate anaerobic conditions. The 96-well plates were then incubated in the chamber of a SpectraMax^®^ 190 microplate reader (Molecular Devices, LLC, United States) at 37°C for 24h. The OD_600_ was measured every 2h to monitor cell growth. After 24h of cultivation, the culture medium was removed, and the biomass in the bottom of the 96-well plates was washed three times with double-distilled water (ddH_2_O) and then air-dried for 2.5h. Next, the biomass was stained with 0.1% safranin solution for 20min, washed five times with ddH_2_O, and air-dried. The safranin-stained biomass was quantified by measuring the OD_450_ value. Biofilm formation is expressed as an index of the biomass stained with safranin (OD_450_ value) normalized to the cell growth (OD_600_ value at 24h). The relative biofilm biomass values were calculated by further normalizing the biofilm index values of the treated group to those of the negative control group to which no *B. subtilis* natto supernatant was added.

### Experimental Setup for Confocal Microscopy

Overnight cultures of *E. faecalis* OG1RF::p23cfp were diluted 1:100 in M9B medium with or without *B. subtilis* natto supernatant treatment (0, 10, and 50% v/v) and cultured on glass coverslips in 35×12mm tissue culture dishes (Alpha Plus Scientific Co., Taiwan). All dishes were incubated anaerobically at 37°C for 24 or 48h. After 24 or 48h of cultivation, the culture medium in the tissue culture dishes was removed, and the biomass attached to the glass coverslips in the tissue culture dishes was washed twice with PBS to remove unattached cells and then fixed with 2% paraformaldehyde (PFA). Fixation with 2% PFA in PBS was performed at 4°C for 10min. After fixation, the biomass attached to the glass coverslip in the bottom of each petri dish was visualized using a white light laser confocal microscope Leica TCS SP8 X (Leica Microsystems, Ltd., Germany) and analyzed using Leica Application Suite X software.

### Extraction and Analysis of Exopolysaccharides From Biofilms

The extraction and analysis of *E. faecalis* biofilm polysaccharides were conducted using the method described by Liu et al. (2020a, 2020b) with some modifications. Overnight cultures of *E. faecalis* were diluted 1:100 in M9B broth with or without *B. subtilis* natto supernatant (0, 10%, or 50% v/v). One milliliter of diluted *E. faecalis* culture was added to 24-well plates and incubated anaerobically at 37°C. After 24 or 48h of cultivation, the culture supernatant was removed, and the biomass that adhered onto the bottom of each well was washed with ddH_2_O and then air-dried for 1h. Next, the adhered biomass was harvested by scraping the surface thoroughly with a sterile polyester-tipped swab after 1ml of PBS was added into each well. The cell suspensions of two wells corresponding to the same treatment were mixed together and centrifuged at 5,000×g for 30min at 4°C. The concentrated precipitates were resuspended in aqueous solution (2ml) containing 0.85% NaCl and 0.22% formaldehyde, and the *E. faecalis* biofilm polysaccharide was extracted at 80°C for 30min. The polysaccharide dissolved in the formaldehyde solution was recovered further *via* centrifugation at 15,000×*g* and 4°C for 30min. The polysaccharide concentrations were quantified using the phenol-sulfuric acid (PSA) method (Dubois et al., 1951). In brief, 100μl of polysaccharide solutions or standard (D-glucose solution) was mixed equally with 5% (w/w) phenol solution in microcentrifuge tubes. Immediately afterward, 1ml of concentrated sulfuric acid was added. The tubes were then incubated for 5min at room temperature, and 200μl of the reaction mixture was added to a 96-well plate. The absorbance was measured at 492nm using a Multiskan FC microplate photometer (Thermo Fisher Scientific Inc., United States).

### HPAEC Analysis of the Carbohydrate Composition of Polysaccharides Obtained From the *E. faecalis* Cell Envelope

*Enterococcus faecalis* cell envelope polysaccharides were prepared using the method described by Dale et al. (2015, 2017) with some modifications. In brief, overnight cultures of *E. faecalis* were diluted 1:100 in M9B broth without and with *B. subtilis* natto supernatant (0 and 10% v/v) and cultured anaerobically. After 24h of incubation, *E. faecalis* cells were collected by centrifugation, and pelleted cells were washed using sucrose solution [25% sucrose and 10mm Tris-HCl (pH 8)]. Cells were then resuspended in sucrose solution supplemented with lysozyme and mutanolysin and incubated overnight at 37°C with gentle agitation. Next, supernatant fractions were harvested by centrifugation, followed by treatment with RNase A, DNase to remove contaminating nucleic acids, and proteinase K to remove protein impurities. The remaining impurities in the supernatant fraction were further extracted with chloroform. The aqueous phase was transferred to a new tube, and polysaccharides were precipitated by the addition of ethanol to a final concentration of 75% and incubation at −80°C for 30min. Precipitated polysaccharides were washed with 75% ethanol and allowed to air dry.

The carbohydrate composition of *E. faecalis* cell envelope polysaccharides was analyzed following complete acid hydrolysis of the polysaccharides. Acid hydrolysis of *E. faecalis* cell envelope polysaccharides was carried out with 1.95N trifluoroacetic acid (TFA) at 80°C for 6h. The mixture was cooled, evaporated, and then resuspended in Milli-Q water. Monosaccharides were analyzed on an HPAEC system (Dionex BioLC) equipped with a gradient pump, a pulsed amperometric detector (PAD) using a gold working electrode, and an anion – exchange column (Carbopac PA −10, 4.6×250mm). The detection condition was an isocratic NaOH concentration of 18mm at ambient temperature. The flow rate was 1.0ml/min. Identification and quantification of the monosaccharides were carried out in comparison with established standards. Data were collected and integrated on a PRIME DAK system (HPLC Technology, Ltd., United Kingdom).

### RNA Purification and Sequencing

Overnight culture of *E. faecalis* OG1RF was diluted 1:100 in M9B medium with or without *B. subtilis* natto supernatant treatment and incubated anaerobically at 37°C for 24h. After 24h of cultivation, 600μl of bacterial culture was treated with 1,200μl of RNAprotect Bacteria Reagent (Qiagen Ltd., Germany) at room temperature for 5min. The cells were then collected by centrifugation for 10min at 4°C, flash-frozen in liquid nitrogen and stored at −80°C until RNA extraction. For RNA extraction, cells were lysed with lysozyme (30mg/ml) and mutanolysin (500U/ml) in Tris-EDTA (TE) buffer at 37°C for 10min (Bandyopadhyay et al., 2016; Manias and Dunny, 2018). Total RNA was then extracted using an RNeasy Mini Kit (Qiagen Ltd., Germany) according to the manufacturer’s instructions. Five micrograms of total RNA was subjected to DNase treatment with Turbo DNase (Ambion Co., United States) according to the manufacturer’s instructions. The RNA purity and concentration were measured using an ND-1000 spectrophotometer (NanoDrop Technologies, Inc., United States), and RNA integrity was validated using a Bioanalyzer 2,100 (Agilent Technologies, Inc., United States). Then, ribosomal RNA was removed using a RiboMinus^™^ Transcriptome Isolation Kit (Invitrogen Co., United States). The cDNA library was constructed with purified mRNA with a SureSelect Strand Specific RNA Library Preparation Kit (Agilent Technologies, Inc., United States) according to the manufacturer’s instructions. RNA-seq using the Illumina NovaSeq 6,000 (paired end) platform was performed at Welgene Biotech (Taiwan).

### RNA-seq Data Analysis

The raw image data were converted to sequence data using bcl2fastq conversion software v2.20. After adaptor clipping and sequence quality trimming with Trimmomatic v0.36, the clean reads of the control and treated groups were mapped to the reference genome of the WT strain *E. faecalis* OG1RF (NC_017316.1) using HISAT2.

Differential expression analysis was performed using cuffdiff (cufflinks v2.2.1) with genome bias detection/correction and Welgene Biotech’s in-house pipeline. Genes with a value of *p*≤0.05 and an FC≥2 were considered significantly differentially expressed. Functional enrichment assays of the differentially expressed genes (DEGs) in each experiment were performed using clusterProfiler v3.6.

### Accession Number(s)

The RNA-seq data discussed in this study have been deposited in NCBI’s Gene Expression Omnibus database (Edgar et al., 2002; Barrett et al., 2013) and are accessible at GEO series accession number GSE184249.^1^

### RT-qPCR

Total RNA was prepared according to the methods above. Approximately 500ng of total RNA was used to synthesize cDNA with a Quant II Fast RT kit (BIOTOOLS Co., Ltd., Taiwan). Each reaction product was diluted 1:20 in ddH_2_O, and 1μl was then used for quantitative real-time polymerase chain reaction (RT-qPCR) using SYBR^®^ Green Supermix (Bio-Rad Laboratories, Inc., United States) and a Bio-Rad CFX384 instrument (Bio-Rad Laboratories, Inc., United States). A total reaction volume of 10μl containing 2μl of gene-specific primer mixture at a concentration of 2μm was used in each well. Each reaction was performed in triplicate, and the threshold cycle (C_T_) values were obtained. Relative quantification was performed using the 2^-ΔΔCT^ method (Livak and Schmittgen, 2001). Gene expression was normalized to that of the housekeeping gene gyrB, which is moderately expressed (Chatterjee et al., 2013). The sequences of the primers are listed in Table 2. Three biological replicates were performed to show repeatability.

**Table 2:** Sequence of the primers used for quantitative real-time polymerase chain reaction (RT-qPCR).

Primer target (name)		Sequence (5′-3′)	Reference
Locus tag	Symbol		
OG1RF_RS00035	gyrB	Forward 5′-CCTATCGGCCTCGGCTTAG-3′ Reverse 5′- AGCGAAAGACAGGTGAGAATCC-3′	Chatterjee et al., 2013
OG1RF_RS01345	copA	Forward 5′-TTAGCCAAAGTGTGCAGCCG-3′ Reverse 5′-CAAGGGACGGATTCAACCGC-3′	This study
OG1RF_RS01350	ctpA	Forward 5′-GCAACAGCGGACCAAGATCG-3′ Reverse 5′- GCAAAGGTCGTCGCATGTGT-3′	This study
OG1RF_RS05035	walK	Forward 5′-TCGCTCGTGGTGATTACGCT-3′ Reverse 5′-CGATCCGTCGCAATGACACC-3′	This study
OG1RF_RS01870	murQ	Forward 5′-GCGATTGCTGCTAGTGGTCG-3′ Reverse 5′-CGCAATCCCAATTTCCGCCA-3′	This study
OG1RF_RS03760	murD	Forward 5′-ACGGGCACCAATGGCAAAAC-3′ Reverse 5′-CTTGAGCCACCGTACTCGCT-3′	This study
OG1RF_RS08680	murN	Forward 5′-CGCCGTCAATGTCGAAAGCA-3′ Reverse 5′-GACGTGGCTGGCATTGTGAA-3′	This study
OG1RF_RS09795	uppS	Forward 5′-TCCAGCAATTCTCGGCAGTCT-3′ Reverse 5′-AAGGCCAAATTCCGCAGCAC-3′	This study
OG1RF_RS11220	bgsA	Forward 5′-CGCATAGGCCCCTTCGATGA-3′ Reverse 5′-TGCGCGACAGTTACCAGAGT-3′	This study
OG1RF_RS12995	guaB	Forward 5′-AACAATCGCATCGGCACCTG-3′ Reverse 5′-CGGTCGTTTACTTGTGGCGG-3′	This study
OG1RF_RS13100		Forward 5′-TTGTTTCCTGTGTTGCCGCC-3′ Reverse 5′-TACCGATGGCGCAGGACATT-3′	This study
OG1RF_RS09175	clpB	Forward 5′-CGGCGTCCATACTGCCTTCT-3′ Reverse 5′-TGCGGGAGCAAAATTCCGTG-3′	This study
OG1RF_RS05115		Forward 5′-TTGTAGCGGTTGGTGTCCGT-3′ Reverse 5′-TCTGGCTCACTTGTCCGCAT-3′	This study
OG1RF_RS06740		Forward 5′-CGTGTCGTTGTCGTTGGTGG-3′ Reverse 5′-ATGCGGTCTAACCCGTCAACT-3′	This study
OG1RF_RS10140	murA	Forward 5′-GCTTCCACTGCTGCTCCTTG-3′ Reverse 5′-GCGTCGAATGAACGCTGACT-3′	This study
OG1RF_RS10725	murB	Forward 5′-AGCGGTTGCTTCAGTTCACG-3′ Reverse 5′- TGCAGAAGTGCTGTTGGCAG −3′	This study
OG1RF_RS03015	murF	Forward 5′- GCAGCGAATGGTGCCGAAAT-3′ Reverse 5′- TGCTGCGCCATTGCTTGATG −3′	This study
OG1RF_RS03765	murG	Forward 5′- TTGACCGGCGCTCGTTTAGT-3′ Reverse 5′- CGCCGTTGCCATCTGTTGTC -3′	This study
OG1RF_RS05030	walR	Forward 5′-CATGGTCTCAAAACGGGGTG-3′ Reverse 5′-AATAACCAACCCCACGACGA-3′	Wu et al., 2021

## Results

### *Bacillus subtilis* natto Supernatant Affects *E. faecalis* Adhesion Onto Human Intestinal Caco-2 Cell Monolayers

**Figure 1:**
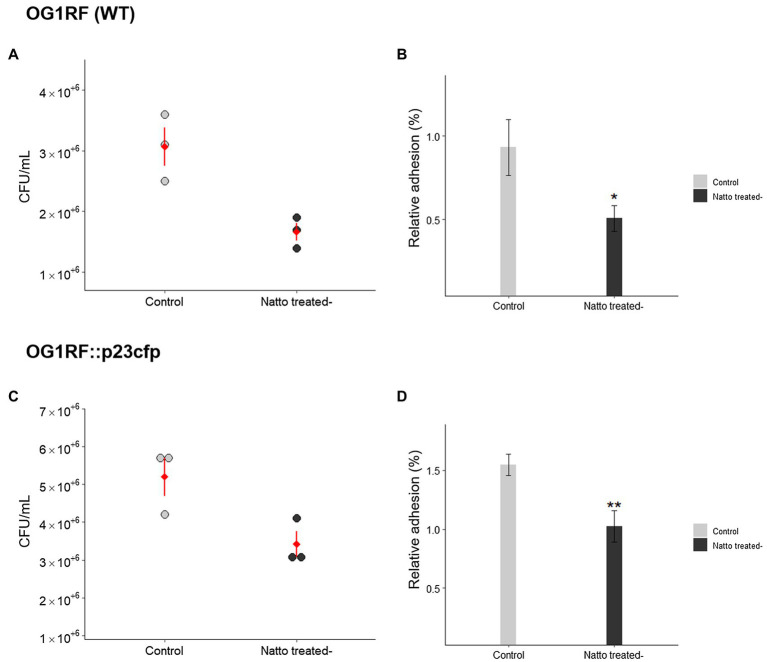
*Bacillus subtilis* natto supernatant affects the adhesion of *Enterococcus faecalis* cells into human intestinal Caco-2 cell monolayers. The concentrations **(A,C)** (CFU/ml) and relative adhesion(%) **(B,D)** of *E. faecalis* OG1RF (WT) and OG1RF::p23cfp cells to Caco-2 cell monolayers after 3h in an *in vitro* bacterial adhesion assay with or without *B. subtilis* natto supernatant treatment (10%, v/v). In **(A)** and **(C)**, each dot in the figures represents a replicate, and the red diamonds with red lines indicate the means±SDs (*n*=3). In **(B)** and **(D)**, the data are presented as the means±SDs (*n*=3). ^*^p<0.05 and ^**^p<0.01 compared with each negative control group (Student’s *t*-test).

Bacterial adhesion to and subsequent colonization of host tissues are key steps in the initial stage of biofilm formation (Baldassarri et al., 2001; Monte et al., 2014). To test our hypothesis that *B. subtilis* natto supernatant would interfere with *E. faecalis* biofilm formation, we first investigated whether the ability of *E. faecalis* to adhere onto the human intestinal epithelial cell line Caco-2 was inhibited by *B. subtilis* natto supernatant treatment. A certain number of *E. faecalis* cells were added to Caco-2 monolayer cultures in the presence or absence of *B. subtilis* natto supernatant. After 3h of cocultivation, the *E. faecalis* cells adhered to the Caco-2 cell monolayers were detached and quantified *via* plating of the entire cultures on selective medium. As shown in Figures 1A,C, the concentrations [colony-forming units (CFU)/ml] of adhered *E. faecalis* cells [both wild-type (WT) and cyan fluorescent protein (CFP)-expressing strains] decreased markedly due to *B. subtilis* natto supernatant treatment. The relative adhesion of *E. faecalis* OG1RF (WT) cells decreased from 1.55 to 1.02%; the relative adhesion of *E. faecalis* OG1RF::p23cfp cells decreased from 0.93 to 0.51% (Figures 1B,D). These results indicated that *B. subtilis* natto supernatant likely interferes with the ability of *E. faecalis* to adhere to host tissues, such as the human intestinal tract.

### *Bacillus subtilis* natto Supernatant Reduces the Formation of *E. faecalis* Aggregates

**Figure 2:**
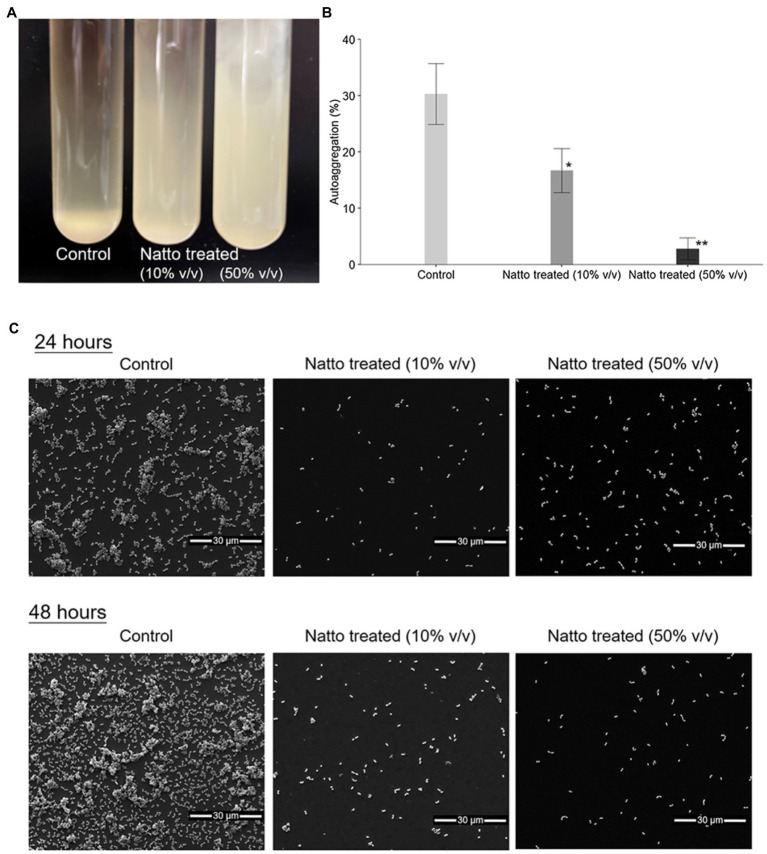
*Bacillus subtilis* natto supernatant inhibits *E. faecalis* autoaggregation. **(A)** Macroscopic analysis of untreated (control) and *B. subtilis* natto supernatant-treated (10 and 50% v/v) *E. faecalis* cultures after 24h of cultivation. **(B)** Percentage autoaggregation exhibited by untreated (control) and *B. subtilis* natto supernatant-treated (10 and 50% v/v) *E. faecalis*. The data are presented as the means±SDs (*n*=3). Values with asterisks (*) were significantly different compared with the negative control (untreated) group according to Duncan’s multiple range tests (^*^p<0.05; ^**^p<0.01). **(C)** SEM images of *E. faecalis* aggregates.

Previous studies (Kragh et al., 2016) have shown that bacterial cells tend to clump together in multicellular aggregates before they form biofilms. Herein, we investigated whether *B. subtilis* natto supernatant affects the formation of *E. faecalis* aggregates. Overnight cultures of *E. faecalis* were diluted in M9B medium in the presence or absence of the *B. subtilis* natto supernatant and incubated under anaerobic conditions. After 24h of cultivation without mixing, both 10 and 50% (v/v) *B. subtilis* natto supernatant-treated *E. faecalis* cultures remained turbid, whereas control (untreated) cultures settled at the bottom of the tube (Figure 2A). Treated and untreated *E. faecalis* cells were collected and used in 4-h autoaggregation assay. As shown in Figure 2B, percentage autoaggregation of both 10 and 50% (v/v) *B. subtilis* natto supernatant-treated *E. faecalis* was significantly lower than that of untreated *E. faecalis* (control group; 10%: *p*<0.05; 50%: *p*<0.01). In addition, the aggregates of *E. faecalis* cells formed on glass coverslips with or without *B. subtilis* natto supernatant were observed and analyzed using scanning electron microscopy (SEM). As shown in Figure 2C, *E. faecalis* cells in the control group formed dense aggregates after 24h of cultivation. However, the dense aggregation of *E. faecalis* cells was markedly reduced upon treatment with 10 and 50% v/v *B. subtilis* natto supernatant. Even though *E. faecalis* grew for 48h, no large cell clumps were observed in the *B. subtilis* natto supernatant-treated groups. Collectively, these results indicated that *B. subtilis* natto supernatant might inhibit *E. faecalis* autoaggregation, thus affecting biofilm initiation and development.

### Inhibition of *E. faecalis* Biofilm Production by *B. subtilis* natto Supernatant

**Figure 3:**
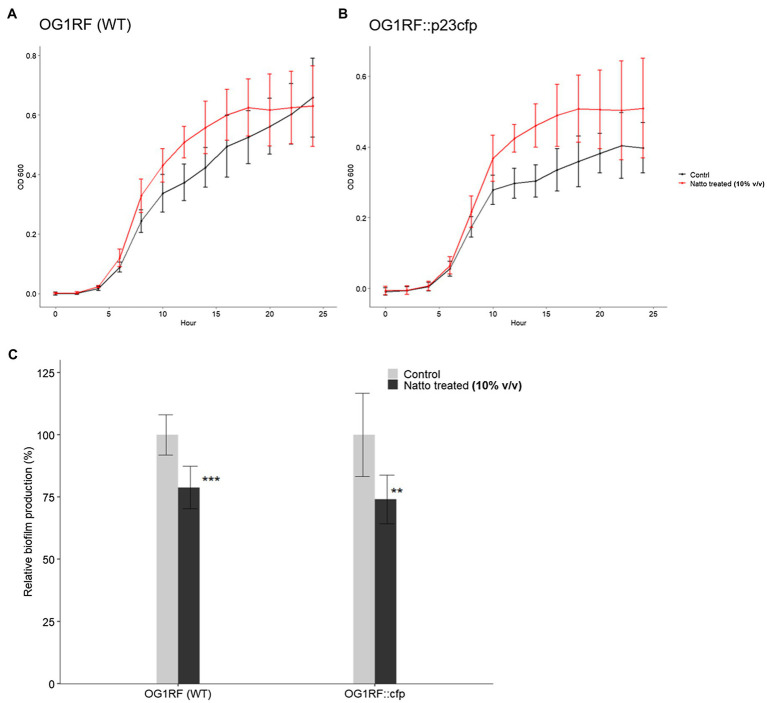
*Bacillus subtilis* natto supernatant interferes with *E. faecalis* biofilm production but not growth. **(A,B)** Growth curves of *E. faecalis* OG1RF WT and OG1RF::p23cfp in the absence or presence of *B. subtilis* natto supernatant (10% v/v) for 24h. In **(A)** and **(B)**, the Y axis shows the OD_600_ values of the bacterial cultures at different time points. The data are presented as the means±SDs (*n*=8). **(C)** Biofilm production in the absence or presence of *B. subtilis* natto supernatant (10% v/v) for 24h. Biofilm production is expressed as an index of the biomass stained with safranin (OD_450_ value) normalized to the cell growth (OD_600_ value at 24h). Relative biofilm production (%) was calculated by further normalizing the biofilm index values of the treated group to those of the negative control group to which no *B. subtilis* natto supernatant was added. The data are presented as the means±SDs (*n*=8). ^**^p<0.01 and ^***^p<0.001 compared with each negative control group (Student’s *t*-test).

Ninety-six-well polystyrene plate-based biofilm assays devised by Dale et al. (2015) were performed here to investigate the effect of *B. subtilis* natto supernatant on *E. faecalis* biofilm growth over 24h. Overnight cultures of *E. faecalis* were diluted in M9B medium in the presence or absence of the *B. subtilis* natto supernatant (10% v/v) and incubated under anaerobic conditions for 24h. The optical density at 600nm (OD_600_) values of the *E. faecalis* cultures were measured every 2h to monitor the growth status of *E. faecalis* within 24h. After 24h of cultivation, the biomass that adhered to the bottom of the 96-well plates was washed, dried, and stained with safranin. Biofilm production was expressed as an index of the biomass stained with safranin [optical density at 450nm (OD_450_) value] normalized to the cell growth (OD_600_ value at 24h). As shown in Figures 3A,B, the OD_600_ values of treated *E. faecalis* cultures were higher than those of untreated *E. faecalis* cultures when *E. faecalis* grew to the exponential growth phase. These results indicated that *B. subtilis* natto supernatant might slightly promote rather than inhibit the growth of *E. faecalis* in 24h. However, the biofilm growth of *E. faecalis* at 24h was significantly inhibited by *B. subtilis* natto supernatant treatment. The relative biofilm production rates (%) of WT and CFP-expressing *E. faecalis* OG1RF strains decreased by 21.18 and 26.99%, respectively (Figure 3C). These results showed that *B. subtilis* natto supernatant could inhibit *E. faecalis* biofilm production without killing planktonic *E. faecalis* cells.

The difference between the *B. subtilis* natto supernatant-treated and untreated groups seemed to be more significant in SEM images (Figure 2C) than in the biofilm formation assay (Figure 3C). In SEM experiments, *E. faecalis* cells attached to glass coverslips that had no coating, whereas *E. faecalis* cells in the biofilm formation assay attached to the tissue culture-treated bottom of a 96 well polystyrene plate. We found that *E. faecalis* cells had relatively difficulty attaching to noncoating abiotic surfaces, such as glass coverslips. Furthermore, some *E. faecalis* cells or clumps that attached to glass coverslips may be removed due to multistep sample pretreatment before SEM observations. Therefore, we inferred that these factors might contribute to the deviation between the results of the SEM experiment and the 96-well plate-based biofilm formation assay (Figures 2C, 3C, respectively).

### *Enterococcus faecalis* Biofilm Architecture Is Impacted by *B. subtilis* natto Supernatant

**Figure 4:**
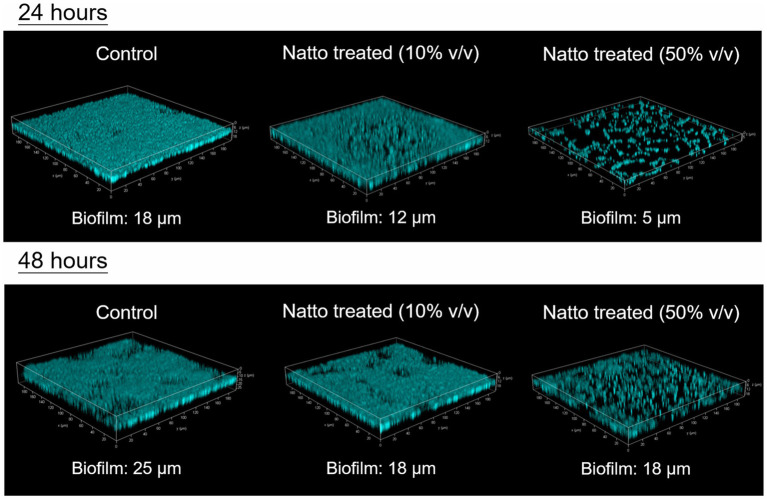
3D architecture of *E. faecalis* OG1RF::p23cfp biofilms with or without *B. subtilis* natto supernatant. The CFP-expressing strain *E. faecalis* OG1RF::p23cfp was cultured anaerobically in M9B medium without or with *B. subtilis* natto supernatant (0, 10, or 50% v/v) on glass coverslips in tissue culture dishes. After 24 or 48h of cultivation, the biofilms that formed on the bottom of the culture dishes were washed, fixed, and then analyzed using CLSM. The approximate biofilm thicknesses (μm) for all groups were measured and are shown in the figure.

The 3D architecture of the biofilms of the CFP- expressing strain *E. faecalis* OG1RF::p23cfp that formed on the glass coverslips at the bottom of the tissue culture dishes with or without *B. subtilis* natto supernatant was visualized and analyzed using confocal laser scanning microscopy (CLSM). The results are shown in Figure 4. After 24h of cultivation, the *E. faecalis* in the control group formed dense and well-organized biofilms, whereas the *E. faecalis* in both *B. subtilis* natto supernatant-treated groups formed loose and even disorganized biofilms, especially those in the group with high-dose *B. subtilis* natto supernatant (50% v/v) treatment. In addition, the biofilms of both 10 and 50% (v/v) *B. subtilis* natto supernatant-treated *E. faecalis* were thinner (at almost 12 and 5μm, respectively) than the *E. faecalis* biofilm in the control group, which was 18μm thick. Although all the control and *B. subtilis* natto supernatant-treated biofilms grew thicker from 24 to 48h, the inhibitory effect of *B. subtilis* natto supernatant on *E. faecalis* biofilms still existed (approximate biofilm thickness: 25μm in the control group, 18μm in both treated groups). The CLSM results were consistent with the results shown by biofilm assays conducted in 96-well polystyrene plates. Taken together, these results indicated that *B. subtilis* natto supernatant interfered with *E. faecalis* biofilm growth and resulted in the formation of looser and thinner biofilms.

### Inhibition of *E. faecalis* Biofilm Polysaccharide Production by *B. subtilis* natto Supernatant

**Figure 5:**
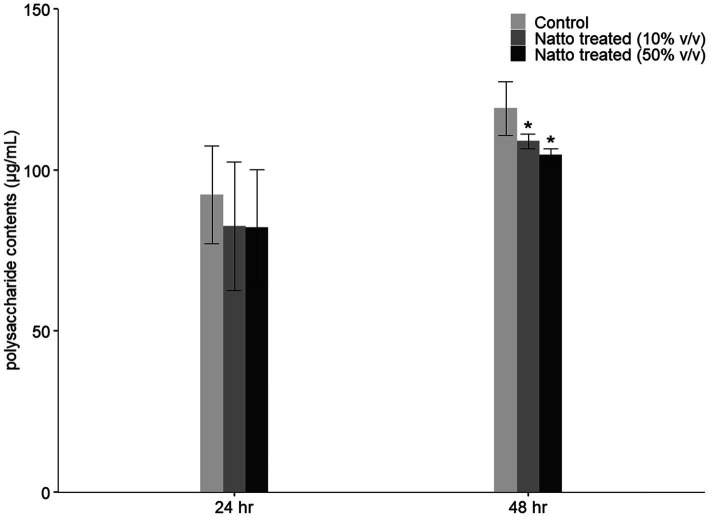
Inhibitory effect of *B. subtilis* natto supernatant on *E. faecalis* biofilm polysaccharide production. *E. faecalis* OG1RF was anaerobically cultured in M9B broth without or with *B. subtilis* natto supernatant (0, 10, and 50% v/v) for 24 or 48h. The polysaccharide contents obtained from *E. faecalis* biofilms were determined using the PSA method. The data are presented as the means±SDs (*n*=3). Values with asterisks (*) were significantly different compared with each negative control group according to Duncan’s multiple range tests (^*^p<0.05).

We found that *E. faecalis* cells can easily and effectively attach to the bottom of tissue culture-treated plates or dishes and form biofilms. Therefore, in the 96-well plate-based biofilm formation assay, we hypothesized that approximately the same number of *E. faecalis* cells attached to the tissue culture-treated surface in the presence or absence of *B. subtilis* natto supernatant. The results in Figure 3C show that *E. faecalis* biofilm production was inhibited by *B. subtilis* natto supernatant treatment. In addition, the *E. faecalis* biofilms in the *B. subtilis* natto supernatant-treated groups were thinner than those in the control (untreated) groups under CLSM (Figure 4). However, *B. subtilis* natto supernatant did not inhibit the growth of *E. faecalis* (Figures 3A,B). Based on these observations, we inferred that the secretion of extracellular polymeric substances (EPSs) in *E. faecalis* biofilms might be changed. When bacteria adhere to a solid surface, they continue to grow and secrete EPS, which comprises polysaccharides, proteins, nucleic acids, and fatty acids (Liu et al., 2020a,b). EPS plays a key role in forming the three-dimensional architecture of biofilms. As reported previously, EPS in most biofilms accounts for more than 90% of the dry mass (Flemming and Wingender, 2010). Furthermore, polysaccharides are the major components of EPS (Flemming and Wingender, 2010). Herein, we investigated whether the polysaccharide contents of *E. faecalis* biofilms were reduced. The quantification results in Figure 5 show that the polysaccharide contents of *E. faecalis* biofilms indeed decreased in both the 10 and 50% (v/v) *B. subtilis* natto supernatant-treated groups after 24h of cultivation. When *E. faecalis* grew for 24 to 48h, the biofilm exopolysaccharide contents in all the control and treated groups increased. However, the polysaccharide contents in both the 10 and 50% (v/v) *B. subtilis* natto supernatant-treated groups were significantly lower than those in the control groups. Thus, *B. subtilis* natto supernatant likely inhibited *E. faecalis* biofilm polysaccharide production.

### *Bacillus subtilis* natto Supernatant Restructures Carbohydrates in the *E. faecalis* Cell Envelope

**Figure 6:**
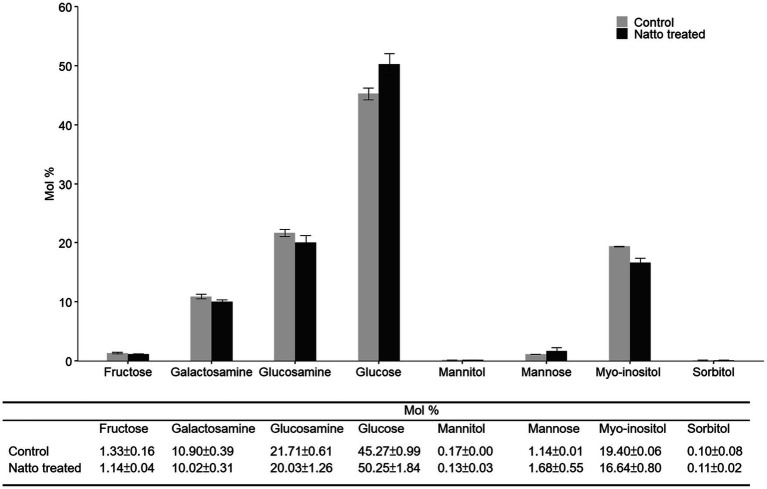
Carbohydrate composition of purified polysaccharides obtained from the *E. faecalis* cell envelope. *E. faecalis* OG1RF was anaerobically cultured in M9B broth without or with *B. subtilis* natto supernatant (0 and 10v/v) for 24h. The polysaccharides in the *E. faecalis* OG1RF cell envelope were purified for carbohydrate composition analysis using HPAEC. The data are presented as the means±SDs (*n*=3).

Previous studies (Haussler et al., 2003; Kragh et al., 2016) have reported that greater cell surface “stickiness” may increase the tendency of bacterial cells to form aggregates and attach to a solid surface, thus increasing biofilm formation. The properties of the bacterial cell surface, including stickiness, may be associated with the composition and organization of the bacterial cell envelope, which comprises the inner cell membrane and the cell wall (Sengupta et al., 2013; Choi et al., 2015). Here, we investigated whether the carbohydrate composition of the *E. faecalis* cell envelope was impacted by *B. subtilis* natto supernatant treatment (10% v/v, 24h) using high-performance anion-exchange chromatography (HPAEC). As shown in Figure 6, several types of monosaccharides, including glucose, glucosamine, and galactosamine, were detected in the *E. faecalis* cell envelope. Glucose, which accounted for approximately 45mol % of all monosaccharides in the *E. faecalis* cell envelope, was the most abundant monosaccharide. Upon treatment with *B. subtilis* natto supernatant, the content of glucose increased by approximately 5mol %, whereas the content of other monosaccharides decreased. Restructuring of the monosaccharide composition in the cell envelope can likely result in changes in cell surface properties, thus affecting the biofilm formation ability of *E. faecalis*.

### RNA-seq Analysis of *E. faecalis* in Response to *B. subtilis* natto Supernatant

**Figure 7:**
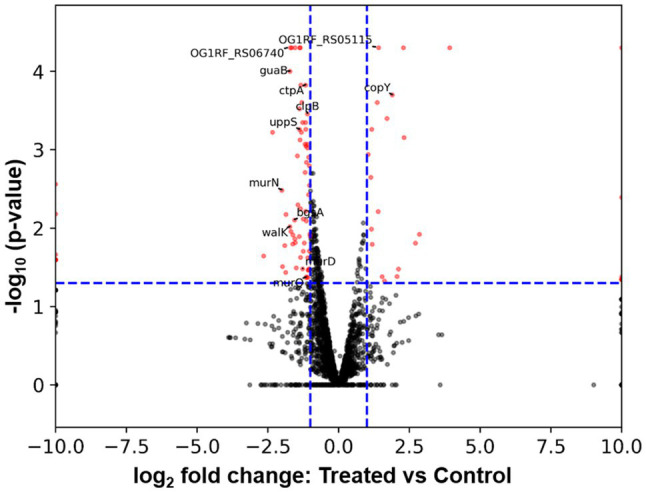
Volcano plot from RNA-seq data showing DEGs of *E. faecalis* in response to *B. subtilis* natto supernatant. The expression of each gene is represented by dots. Genes with a value of *p*≤0.05 and an FC≥2 were considered significantly differentially expressed (represented by red dots). The horizontal blue dashed lines indicate the positions on the Y axes for a -log_10_ (value of *p*=0.05), and the vertical blue dashed lines indicate the positions on the X axes for a±log_2_ (FC=2). The 13 DEGs labeled in the volcano plot were selected for further RT-qPCR analysis.

To further understand how *E. faecalis* responded to *B. subtilis* natto supernatant, we searched for DEGs between *E. faecalis* treated with or without *B. subtilis* natto supernatant using RNA-seq analysis. According to the RNA-seq results, approximately 95.94 and 97.98% of the clean reads from the treated and control (untreated) groups were mapped to the reference genome, respectively. Among the 2,657 genes detected by RNA-seq [2,658 genes present in *E. faecalis* OG1RF (Bourgogne et al., 2008)], 95 genes were identified as differentially expressed [value of *p*≤0.05 and fold change (FC)≥2] in the treated group (Figure 7 and Supplementary Table 1). Among these DEGs, 70 genes were identified as significantly downregulated, and 25 genes were found to be significantly upregulated. Furthermore, Gene Ontology (GO) annotation analysis and Kyoto Encyclopedia of Genes and Genomes (KEGG) pathway enrichment analysis were performed to identify DEGs at the biological function level. The GO annotation results showed that the genes associated with the ATP binding term were differentially expressed, and the KEGG pathway mapping results showed that some DEGs were involved in the peptidoglycan biosynthetic process (Supplementary Figures 1, 2). These results indicated that some genes encoding ATP binding protein or peptidoglycan biosynthetic genes might be differentially expressed due to *B. subtilis* natto supernatant treatment.

To verify the RNA-seq results, we selected a total of 13 DEGs, including some ATP binding protein-encoding genes and peptidoglycan biosynthetic genes, for further RT-qPCR analysis. As listed in Table 3, the up- or downregulation trends and relative expression of most selected DEGs, including three peptidoglycan biosynthetic genes [murD (OG1RF_RS03760), murN (OG1RF_RS08680), and uppS (OG1RF_RS09795)], bgsA (OG1RF_RS11220), and walK (OG1RF_RS05035), were consistent with the RNA-seq results. Furthermore, a correlation between the six downregulated DEGs listed above and *E. faecalis* cell wall homeostasis and biofilm formation has been reported previously (Dubrac et al., 2008; Vollmer et al., 2008; Theilacker et al., 2009; Watanabe et al., 2012; Bucher et al., 2015). Peptidoglycan, an essential component of the cell wall in almost all bacteria, plays a key role in maintaining cell shape and serves as a scaffold to anchor other cell envelope components, such as proteins and teichoic acids (Vollmer et al., 2008). Gene bgsA (OG1RF_RS11220) encodes the putative glucosyltransferase designated biofilm-associated glycolipid synthesis A and synthesizes diglucosyl–diacylglycerol (DGlcDAG) in *E. faecalis* (Theilacker et al., 2009). DGlcDAG, a precursor of glycolipid and lipoteichoic acid, is involved in *E. faecalis* biofilm production, adherence to host cells and virulence *in vivo* (Theilacker et al., 2009). Gene walK (OG1RF_RS05035) encodes the membrane-linked histidine kinase WalK. WalK is a key element in the WalK/WalR two-component signal transduction system, which regulates genes involved in cell wall metabolism, biofilm production, virulence regulation, oxidative stress resistance, and antibiotic resistance in low-G+C Gram-positive bacteria, including *E. faecalis* (Dubrac et al., 2008; Watanabe et al., 2012). Based on these findings, we focused on the six downregulated cell wall- and biofilm-related genes in *E. faecalis* in further investigations.

**Table 3:** Validation of RNA-seq results by RT-qPCR.

Locus tag	Symbol	Annotation	FC (treated/control)	
			^a^RNA-seq	RT-qPCR
OG1RF_RS01345	copY	CopY/TcrY family copper transport repressor	3.73	0.32±0.05
OG1RF_RS01350	ctpA	copper-translocating P-type ATPase	0.45	0.26±0.01
OG1RF_RS01870	murQ	N-acetylmuramic acid 6-phosphate etherase	0.45	0.41±0.11
OG1RF_RS03760	murD	UDP-N-acetylmuramoyl-L-alanine--D-glutamate ligase	0.41	0.80±0.15
OG1RF_RS05035	walK	cell wall metabolism sensor histidine kinase WalK	0.30	0.49±0.03
OG1RF_RS05115		NADH peroxidase	2.67	1.03±0.19
OG1RF_RS06740		SidA/IucD/PvdA family monooxygenase	0.31	0.25±0.15
OG1RF_RS08680	murN	aminoacyltransferase	0.25	0.25±0.01
OG1RF_RS09175	clpB	ATP-dependent chaperone ClpB	0.47	0.86±0.19
OG1RF_RS09795	uppS	isoprenyl transferase	0.38	0.41±0.03
OG1RF_RS12995	guaB	IMP dehydrogenase	0.30	0.58±0.09
OG1RF_RS13100		cell wall surface anchor protein	2.25	1.00±0.29
OG1RF_RS11220	bgsA	cell wall glycolipid biosynthesis glucosyltransferase BgsA	0.34	0.32±0.09

a *Relative gene expression normalized to gyrB. The 2^−ΔΔCT^ method was used for calculations*.

### Inhibitory Effect of *B. subtilis* natto Supernatant on the *E. faecalis* Peptidoglycan Biosynthesis Pathway and WalK/WalR Two-Component System

**Figure 8:**
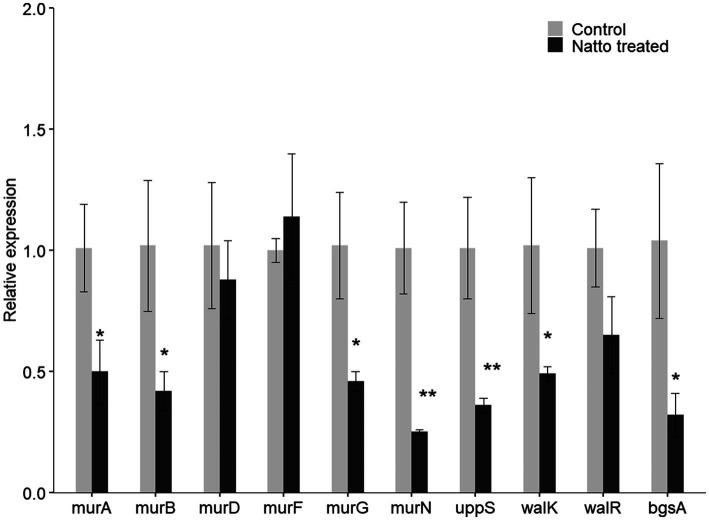
*B. subtilis* natto supernatant inhibits *E. faecalis* cell wall- and biofilm-related genes. *E. faecalis* OG1RF was cultured anaerobically in the presence or absence of *B. subtilis* natto supernatant for 24h. The relative expression of genes in the peptidoglycan biosynthesis pathway (murA, murB, murD, murF, and uppS), genes in the WalK/WalR two-component system (walK and walR), and bgsA were assessed using RT-qPCR. The data are presented as the means±SDs (*n*=3). ^*^p<0.05 and ^**^p<0.01 compared with each negative control group (Student’s *t*-test).

To investigate whether *B. subtilis* natto supernatant inhibits the expression of other genes in the peptidoglycan biosynthesis pathway and WalK/WalR regulatory system, the relative expression of UDP-GlcNAc enolpyruvyl transferase (murA; OG1RF_RS10140), murB (OG1RF_RS10725), murF (OG1RF_RS03015), and murG (OG1RF_RS03765) in the peptidoglycan biosynthesis pathway and the response regulator-encoding gene walR (OG1RF_RS05030) in the WalK/WalR two-component system were assessed further using RT-qPCR. As shown in Figure 8, the relative expression of several peptidoglycan biosynthetic genes except murF was indeed inhibited due to *B. subtilis* natto supernatant treatment. The relative expression of walR in the WalK/WalR two-component system was also inhibited. Taken together with the RT-qPCR results of the six cell wall- and biofilm-related genes identified from RNA-seq, these results indicate that *B. subtilis* natto supernatant might interfere with the *E. faecalis* peptidoglycan biosynthesis pathway, WalK/WalR two-component system and bgsA, thus inhibiting biofilm formation.

## Discussion

In this study, we found that the culture supernatant of *B. subtilis* natto can potently inhibit *E. faecalis* adherence to Caco-2 cell monolayers, aggregation, and biofilm production. These findings support the idea that some derivatives present in *B. subtilis* natto supernatant may have antibiofilm activity against *E. faecalis*. Consistent with this idea, some derivatives of *Bacillus* species have been shown to inhibit the formation of biofilms by several bacterial pathogens (Rivardo et al., 2009; Yoo et al., 2019; Tazehabadi et al., 2021). Rivardo et al. (2009) found that biosurfactants secreted by *B. subtilis* and *B. licheniformis* can effectively inhibit biofilm formation of *Escherichia coli* and *S. aureus*. Yoo et al. (2019) reported that *Bacillus velezensis* supernatant containing 1-deoxynojirimycin can decrease the biofilm production of *Streptococcus mutans*. Tazehabadi et al. (2021) mentioned that the bacteriocins subtilosin A and subtilin produced by *B. subtilis* and *B. amyloliquefaciens*, respectively, play a key role in inhibiting *Salmonella* biofilm formation (Yoo et al., 2019).

We also found that *E. faecalis* produced fewer exopolysaccharides under treatment with the culture supernatant of *B. subtilis* natto than under control conditions. Previous studies (Ramos et al., 2019) have shown that β-1,6-linked poly-N-acetylglucosamine (polyGlcNAc) in exopolysaccharides produced by *E. faecalis* bacteria enables the bacteria to successfully penetrate semisolid surfaces and translocate through human epithelial cell monolayers (Ramos et al., 2019). Thus, the inhibitory effect on *E. faecalis* exopolysaccharide production might also contribute to reducing polyGlcNAc production, thus mitigating the pathogenicity of *E. faecalis*.

In addition to exerting an inhibitory effect on exopolysaccharide production, *B. subtilis* natto supernatant restructured carbohydrates in the *E. faecalis* cell envelope. A previous study reported that the structure and composition of the bacterial cell envelope are linked to bacterial cell surface properties, such as surface stickiness (Sengupta et al., 2013). Another study reported that bacterial cells with greater surface stickiness have a tendency to aggregate (Kragh et al., 2016). In addition, the increased tendency to aggregate has been shown to be associated with increased biofilm production in *Pseudomonas aeruginosa* (Deziel et al., 2001; Haussler et al., 2003). Based on these findings, we infer that *B. subtilis* natto supernatant may alter *E. faecalis* cell envelope composition and cell surface properties, thereby interfering with *E. faecalis* adhesion to host tissues, aggregation, and biofilm production.

*Via* RNA-seq and RT-qPCR analysis, we found that *B. subtilis* natto supernatant inhibited the expression of bgsA, which encodes biofilm-associated glycolipid synthesis A, in *E. faecalis*. Previous studies (Theilacker et al., 2009) have shown that inactivation of bgsA in *E. faecalis* leads to a lack of DGlcDAG in cell membranes and to accumulation of longer lipoteichoic acid structures in cell walls, thus impairing *E. faecalis* adherence to host tissues and biofilm production. In addition to bgsA, walK and walR in the WalK/WalR two-component system were inhibited in *E. faecalis* treated with *B. subtilis* natto supernatant. In low-G+C Gram-positive bacteria, the activation of the WalK/WalR two-component system is essential for lateral cell wall synthesis and cell elongation, whereas depletion of this system may cause defects in cell morphology, murein (peptidoglycan) synthesis, and biofilm formation (Dubrac et al., 2008; Takada and Yoshikawa, 2018). Moreover, we also found that several peptidoglycan biosynthetic genes (murA, murB, murD, murG, murN, and uppS) in *E. faecalis* were inhibited. Since peptidoglycan is a crucial structural element in the cell walls of most bacteria, interference with its biosynthesis results in impaired biofilm formation and even cell lysis (Vollmer et al., 2008; Bucher et al., 2015). The downregulated genes listed above are all related to cell envelope synthesis and biofilm formation in *E. faecalis*. The evidence suggests that *B. subtilis* natto supernatant targets *E. faecalis* cell envelope synthesis and therefore interferes with the cell envelope composition and biofilm formation of *E. faecalis*.

Our findings reveal that *B. subtilis* natto supernatant can likely inhibit biofilm formation of *E. faecalis via* interference with *E. faecalis* cell envelope synthesis. Notably, the bacterial cell envelope synthetic process has been reported to be the major target for many antibacterial agents (McCallum et al., 2011). Many antibiotics act by blocking or disrupting bacterial peptidoglycan biosynthesis, such as fosfomycin, which inhibits MurA (Hashemian et al., 2019), and tunicamycin, which inhibits bacterial phospho-N-acetylmuramic acid (MurNAc)-pentapeptide translocase (MraY; Yamamoto et al., 2019). Furthermore, because maintenance of cell wall homeostasis and growth in low-G+C Gram-positive bacteria is essential, the WalK/WalR two-component system has been proposed as a novel target for antibacterial agents that are effective against multidrug-resistant bacteria, including MRSA and vancomycin-resistant *E. faecalis* (Fabret and Hoch, 1998; Gotoh et al., 2010). Watanabe et al. (2012) reported that the novel antibiotic signermycin B from *Streptomyces* extracts can function as a WalK inhibitor, targeting the conserved dimerization domain of WalK to inhibit autophosphorylation (Watanabe et al., 2012). Collectively, these studies provide evidence that some substances that function like antibiotics or WalK inhibitors may be present in the culture supernatant of *B. subtilis* natto. Further investigations are necessary to identify potential antibiofilm agents in the culture supernatant of *B. subtilis* natto.

The therapeutic potential of *B. subtilis* and its derivatives in animals has been reported in previous studies (Cartman et al., 2008; Piewngam et al., 2018). One previous study (Cartman et al., 2008) showed that *B. subtilis* spores can germinate in the chicken gastrointestinal tract. Another study (Piewngam et al., 2018) showed that mice fed *B. subtilis* spores exhibit complete decolonization of MRSA in the feces and intestines. These studies provide evidence that *B. subtilis* natto spores may germinate to form vegetative cells and produce functional substances in host gastrointestinal tracts.

In this work, our results showed that *B. subtilis* natto derivatives present in the culture supernatant could effectively inhibit the formation of *E. faecalis* biofilms. These derivatives downregulated the transcription of genes involved in membrane glycolipid biosynthesis, the WalK/WalR two-component system, and peptidoglycan biosynthesis, which may contribute to changes in the structural components of the cell envelope and therefore affect biofilm formation ability in *E. faecalis*. Based on these findings, we propose that natto or the probiotic *B. subtilis* natto could be used in the management of *E. faecalis* biofilm infections.

## Data Availability Statement

The datasets presented in this study can be found in online repositories. The names of the repository/repositories and accession number(s) can be found online at: https://www.ncbi.nlm.nih.gov/geo/, GSE184249.

## Author Contributions

Y-CL wrote the manuscript. K-TL, W-SH, and Y-CL designed the experimental plan. Y-CL and C-YW performed all experiments and analyzed the relevant data. H-TH and M-KL assisted with carbohydrate composition analysis using HPAEC and data interpretation. All authors contributed to the revision and final review of the manuscript.

## Funding

This work was funded by grants from the Ministry of Science and Technology (nos. 107-2313-B-002-028 and 108-2313-B-002-056-MY3), Taiwan.

## Conflict of Interest

The authors declare that the research was conducted in the absence of any commercial or financial relationships that could be construed as a potential conflict of interest.

## Publisher’s Note

All claims expressed in this article are solely those of the authors and do not necessarily represent those of their affiliated organizations, or those of the publisher, the editors and the reviewers. Any product that may be evaluated in this article, or claim that may be made by its manufacturer, is not guaranteed or endorsed by the publisher.

## Footnotes

^1^ https://www.ncbi.nlm.nih.gov/geo/query/acc.cgi?acc=GSE184249

## Supplementary Material

The Supplementary Material for this article can be found online at:

https://www.frontiersin.org/articles/10.3389/fmicb.2021.785351/full#supplementary-material

## Abbreviations

MRSA: methicillin-resistant *Staphylococcus aureus*
γ-PGA: γ-poly-DL-glutamic acid
FBS: fetal bovine serum
PFA: paraformaldehyde
EPS: extracellular polymeric substance
PSA: phenol-sulfuric acid
HPAEC: high-performance anion-exchange chromatography
PAD: pulsed amperometric detector
TFA: trifluoroacetic acid
TE: Tris-EDTA
PBS: phosphate-buffered saline
A: absorbance
SEM: scanning electron microscopy
CLSM: confocal laser scanning microscopy
rRNA: ribosomal RNA
qRT-qPCR: quantitative real-time polymerase chain reaction
OD: optical density
ddH2O: double-distilled water
CFU: colony-forming units
WT: wild type
CFP: cyan fluorescent protein
DEGs: differentially expressed genes
FC: fold change
1-DNJ: 1-deoxynojirimycin
polyGlcNAc: β-1,6-linked poly-N-acetylglucosamine
