# Supplementary material for: Bacillus subtilis natto Derivatives Inhibit Enterococcal Biofilm Formation via Restructuring of the Cell Envelope
Source: Front Microbiol. 2021 Dec 9;12:785351. doi: 10.3389/fmicb.2021.785351 (PMC8695906; doi:10.3389/fmicb.2021.785351)
Supplement: Supplementary file 1 [file Data_Sheet_1.PDF]

Supplementary Material

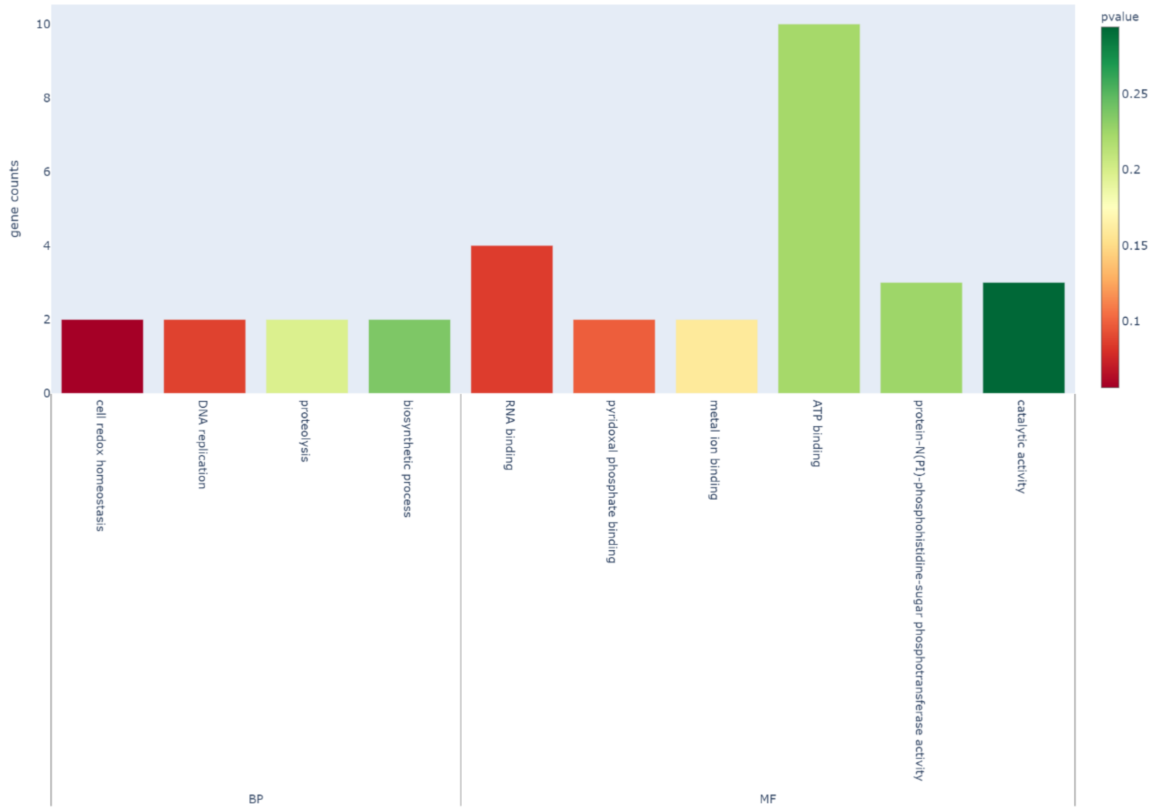

Supplementary Figure 1. Bar plot of the GO enrichment results for the DEGs. Each bar represents a GO term (top 10). The top 10 GO terms were mainly divided into two categories: the biological process (BP) and molecular function (MF) categories. The X-axis shows the GO term classification, and the y-axis shows the number of genes. The color indicates the significance of enrichment (p-value), with the color gradient representing the degree of the p-value.

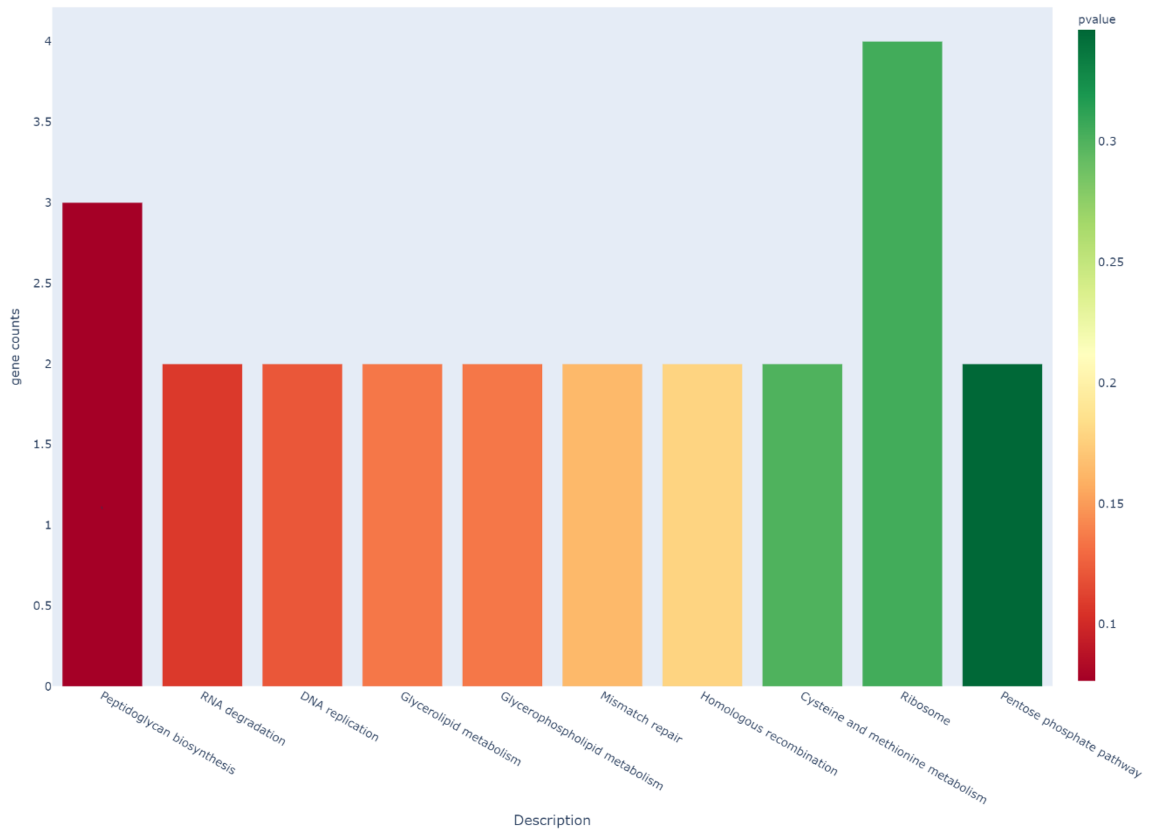

Supplementary Figure 2. Bar plot of the KEGG pathways of the DEGs. Each bar represents a pathway (top 10). The X-axis shows the pathway classification, and the y-axis shows the number of genes. The color indicates the significance of enrichment (p-value), with the color gradient representing the degree of the p-value.

Supplementary Table 1. A total of 95 DEGs (p value ≤ 0.05 and FC ≥ 2) were identified in the *B. subtilis* natto supernatant-treated group.

Locus tag	Symbol	Annotation	FPKM		Ratio & log_2 ratio		P-value
			Treated	Control	T/C	log_2(T/C)	
OG1RF_RS00090		sigma-54-dependent transcriptional regulator	4.25	10.47	0.41	-1.30	0.00025
OG1RF_RS00125		hypothetical protein	203.28	470.91	0.43	-1.21	0.0155
OG1RF_RS00140		pyridoxal phosphate-dependent aminotransferase	1.06	0.25	4.33	2.12	0.0333
OG1RF_RS01020		hypothetical protein	30.29	1.00E-05	3.03E+06	21.53	0.04525
OG1RF_RS01195		tRNA-Ser	3445.22	1.00E-05	3.45E+08	28.36	0.04525
OG1RF_RS01345	copY	CopY/TcrY family copper transport repressor	23.78	6.38	3.73	1.90	0.0002
OG1RF_RS01350	ctpA	copper-translocating P-type ATPase	27.94	62.70	0.45	-1.17	0.00015
OG1RF_RS01575		DUF969 domain-containing protein	14.94	31.44	0.48	-1.07	0.0346
OG1RF_RS01625		type I toxin-antitoxin system Fst family toxin	904.15	276.32	3.27	1.71	0.0004
OG1RF_RS01800	menB	1,4-dihydroxy-2-naphthoyl-CoA synthase	27.70	59.74	0.46	-1.11	0.00085
OG1RF_RS01870	murQ	N-acetylmuramic acid 6-phosphate etherase	1.18	2.63	0.45	-1.15	0.0425
OG1RF_RS02105		uracil-DNA glycosylase	347.82	71.11	4.89	2.29	0.00005
OG1RF_RS02520		nicotinamide riboside transporter PnuC	9.44	21.14	0.45	-1.16	0.0061
OG1RF_RS02545		L-cystine transporter	5.66	11.66	0.49	-1.04	0.03305
OG1RF_RS03150		copper-translocating P-type ATPase	42.56	88.54	0.48	-1.06	0.00125
OG1RF_RS03595		hypothetical protein	0.00	2.66	0.00	-18.02	0.02535
OG1RF_RS03740	rsmH	16S rRNA (cytosine(1402)-N(4))-methyltransferase RsmH	0.73	2.06	0.35	-1.50	0.0322
OG1RF_RS03760	murD	UDP-N-acetylmuramoyl-L-alanine--D-glutamate ligase	0.66	1.59	0.41	-1.27	0.0331
OG1RF_RS03800		RNA-binding protein	15.13	33.82	0.45	-1.16	0.00055
OG1RF_RS03885		PTS sugar transporter subunit IIC	31.63	14.35	2.20	1.14	0.00225
OG1RF_RS03900		hypothetical protein	2.51E+05	1.22E+05	2.07	1.05	0.00115
OG1RF_RS03990		Cof-type HAD-IIB family hydrolase	3.49	10.73	0.33	-1.62	0.01595
OG1RF_RS03995		DUF1361 domain-containing protein	0.00	0.59	0.00	-15.84	0.02535
OG1RF_RS04060		16S ribosomal RNA	124.51	8.20	15.19	3.93	0.00005
OG1RF_RS04075	rrf	5S ribosomal RNA	4508.43	1.00E-05	4.51E+08	28.75	0.00005
OG1RF_RS04340		hypothetical protein	4.38	13.84	0.32	-1.66	0.00005
OG1RF_RS04590		DUF898 family protein	0.00	2.88	0.00	-18.13	0.02535
OG1RF_RS04650	lepB	signal peptidase I	13.79	35.04	0.39	-1.35	0.00565
OG1RF_RS04675		phenylalanine--tRNA ligase subunit beta	27.76	71.24	0.39	-1.36	0.00005
OG1RF_RS05010		TIGR01906 family membrane protein	13.93	2.80	4.97	2.31	0.0007

OG1RF_RS05035	walK	cell wall metabolism sensor histidine kinase WalK	1.04	3.42	0.30	-1.72	0.0096
OG1RF_RS05050		MBL fold metallo-hydrolase	15.11	31.50	0.48	-1.06	0.00375
OG1RF_RS05060		TIGR03943 family protein	8.36	3.74	2.23	1.16	0.0103
OG1RF_RS05115		NADH peroxidase	1502.30	563.36	2.67	1.42	0.00005
OG1RF_RS05460		hypothetical protein	2.21	1.00E-05	2.21E+05	17.76	0.04195
OG1RF_RS05470		structural protein	43.93	19.28	2.28	1.19	0.0161
OG1RF_RS05845		hypothetical protein	0.73	1.00E-05	7.33E+04	16.16	0.04525
OG1RF_RS06085		molybdenum cofactor guanylyltransferase	1.32	1.00E-05	1.32E+05	17.01	0.00405
OG1RF_RS06200		ABC transporter ATP-binding protein	2.12	6.83	0.31	-1.68	0.01105
OG1RF_RS06245		hypothetical protein	978.78	2547.60	0.38	-1.38	0.0003
OG1RF_RS06250	nrdI	class Ib ribonucleoside-diphosphate reductase assembly flavoprotein NrdI	8.63	2.06	4.18	2.07	0.04145
OG1RF_RS06520		ECF transporter S component	82.76	183.94	0.45	-1.15	0.0009
OG1RF_RS06705	lexA	transcriptional repressor LexA	3.88	11.85	0.33	-1.61	0.01225
OG1RF_RS06740		SidA/IucD/PvdA family monooxygenase	481.57	1565.70	0.31	-1.70	0.00005
OG1RF_RS06750		GNAT family N-acetyltransferase	1.00E-05	2.67	0.00	-18.03	0.0066
OG1RF_RS06780		NUDIX domain-containing protein	1.00E-05	1.29	0.00	-16.98	0.02175
OG1RF_RS06875	parE	DNA topoisomerase IV subunit B	8.15	17.89	0.46	-1.13	0.00145
OG1RF_RS07250	rpsP	30S ribosomal protein S16	59.91	125.21	0.48	-1.06	0.03395
OG1RF_RS07275		putative DNA-binding protein	17.59	44.64	0.39	-1.34	0.02365
OG1RF_RS07295	pstS	phosphate ABC transporter substrate-binding protein PstS family protein	2.49	6.41	0.39	-1.36	0.00075
OG1RF_RS07545		phosphate ABC transporter ATP-binding protein	9.17	19.50	0.47	-1.09	0.0239
OG1RF_RS07690		phosphoribosylaminoimidazolesuccinocarboxamide synthase	1.68	8.48	0.20	-2.33	0.0006
OG1RF_RS07740		DUF998 domain-containing protein	1.44	3.78	0.38	-1.39	0.0128
OG1RF_RS08050		TVP38/TMEM64 family protein	4.25	12.30	0.35	-1.53	0.01535
OG1RF_RS08115		lactonase family protein	6.79	2.34	2.90	1.54	0.04165
OG1RF_RS08345		SorC family transcriptional regulator	81.49	183.25	0.44	-1.17	0.00045
OG1RF_RS08350		YitT family protein	1.90	4.52	0.42	-1.25	0.00765
OG1RF_RS08580	mnmA	tRNA 2-thiouridine(34) synthase MnmA	23.18	58.46	0.40	-1.33	0.00015
OG1RF_RS08680	murN	aminoacyltransferase	0.91	3.65	0.25	-2.01	0.0033
OG1RF_RS08925		TetR/AcrR family transcriptional regulator	4.07	0.56	7.24	2.86	0.012
OG1RF_RS08990		FUSC family protein	1.02	4.03	0.25	-1.98	0.031
OG1RF_RS09105		hypothetical protein	24.11	9.36	2.58	1.37	0.00025
OG1RF_RS09175	clpB	ATP-dependent chaperone ClpB	884.72	1898.79	0.47	-1.10	0.00035

OG1RF_RS09305	rseP	RIP metalloprotease RseP	1.72	3.76	0.46	-1.13	0.0421
OG1RF_RS09325		ATP-binding protein	5.74	13.05	0.44	-1.18	0.00195
OG1RF_RS09330		methyltransferase	0.89	5.56	0.16	-2.65	0.0227
OG1RF_RS09485		homoserine dehydrogenase	22.87	46.72	0.49	-1.03	0.00285
OG1RF_RS09550		PTS transporter subunit EIIC	3.72	1.41	2.64	1.40	0.00615
OG1RF_RS09605	holA	DNA polymerase III subunit delta	3.73	10.11	0.37	-1.44	0.00505
OG1RF_RS09675	recQ	DNA helicase RecQ	2.67	5.89	0.45	-1.14	0.0081
OG1RF_RS09685		inositol monophosphatase family protein	60.22	123.47	0.49	-1.04	0.0016
OG1RF_RS09790		phosphatidate cytidylyltransferase	4.81	17.99	0.27	-1.90	0.0167
OG1RF_RS09795	uppS	isoprenyl transferase	16.27	42.43	0.38	-1.38	0.00055
OG1RF_RS09850		hypothetical protein	3.62	7.31	0.50	-1.01	0.0119
OG1RF_RS09995		purine permease	5.37	10.79	0.50	-1.01	0.0127
OG1RF_RS10200	rnr	ribonuclease R	14.24	35.46	0.40	-1.32	0.0006
OG1RF_RS10290		ABC-F family ATP-binding cassette domain-containing protein	25.61	74.25	0.34	-1.54	0.00005
OG1RF_RS10475	trpS	tryptophan--tRNA ligase	2.90	8.46			0.01375
OG1RF_RS10535		1-acyl-sn-glycerol-3-phosphate acyltransferase	9.33	25.60			0.0012
OG1RF_RS10860	holB	DNA polymerase III subunit delta'	48.87	104.54			0.00095
OG1RF_RS11175	fabG	3-oxoacyl-[acyl-carrier-protein] reductase	53.14	120.93			0.00085
OG1RF_RS11220	bgsA	glycosyltransferase	3.53	10.36			0.0079
OG1RF_RS11230	rrf	5S ribosomal RNA	4.73E+04	1.22E+05			0.00005
OG1RF_RS11255		hypothetical protein	36.81	75.53			0.01965
OG1RF_RS11450	thiI	tRNA 4-thiouridine(8) synthase ThiI	1.51	3.58			0.01975
OG1RF_RS11525		hypothetical protein	1.00E-05	1.88			0.02535
OG1RF_RS11545		amidinotransferase	13.64	32.91			0.00045
OG1RF_RS11580		PTS sugar transporter subunit IIB	1.00E-05	2.61			0.00275
OG1RF_RS11785		dicarboxylate/amino acid:cation symporter	0.78	2.85			0.0067
OG1RF_RS12535		WxL domain-containing protein	3.26	0.50			0.01555
OG1RF_RS12625		zinc ABC transporter substrate-binding protein AdcA	1.55	3.29			0.04265
OG1RF_RS12910		aminotransferase class I/II-fold pyridoxal phosphate-dependent enzyme	0.77	2.82			0.0368
OG1RF_RS12995	guaB	IMP dehydrogenase	4.49	14.80			0.0001
OG1RF_RS13100		cell wall surface anchor protein	7.42	3.30			0.00055
OG1RF_RS13840		hypothetical protein	50.47	16.38			0.0474
